# Malignant cancer and invasive placentation: A case for positive pleiotropy between endometrial and malignancy phenotypes

**DOI:** 10.1093/emph/eou022

**Published:** 2014-10-15

**Authors:** Alaric W. D'Souza, Günter P. Wagner

**Affiliations:** ^1^Department of Ecology and Evolutionary Biology, Yale University, New Haven, CT, USA; ^2^Systems Biology Institute, Yale University, New Haven, CT, USA; ^3^Department of Obstetrics and Gynecology, Wayne State University, Detroit, MI, USA

**Keywords:** evolution of malignancy, evolution of placentation, antagonistic pleiotropy, cancer

## Abstract

Cancer metastasis is an invasive process that involves the transplantation of cells into new environments. Since human placentation is also invasive, hypotheses about a relationship between invasive placentation in eutherian mammals and metastasis have been proposed. The relationship between metastatic cancer and invasive placentation is usually presented in terms of antagonistic pleiotropy. According to this hypothesis, evolution of invasive placentation also established the mechanisms for cancer metastasis. Here, in contrast, we argue that the secondary evolution of less invasive placentation in some mammalian lineages may have resulted in positive pleiotropic effects on cancer survival by lowering malignancy rates. These positive pleiotropic effects would manifest themselves as resistance to cancer cell invasion. To provide a preliminary test of this proposal, we re-analyze data from Priester and Mantel (Occurrence of tumors in domestic animals. Data from 12 United States and Canadian colleges of veterinary medicine. *J Natl Cancer Inst* 1971;**47**:1333-44) about malignancy rates in cows, horses, cats and dogs. From our analysis we found that equines and bovines, animals with less invasive placentation, have lower rates of metastatic cancer than felines and canines in skin and glandular epithelial cancers as well as connective tissue sarcomas. We conclude that a link between type of placentation and species-specific malignancy rates is more likely related to derived mechanisms that suppress invasion rather than different degrees of fetal placental aggressiveness.

## INTRODUCTION

Tumors are an inescapable consequence of senescence [1, 2]. Over time, somatic mutations accumulate, and selection pressures ensure that cells with fast division times and selfish resource tendencies emerge [3]. The true danger from tumors, however, lies in metastasis, i.e. when tumor cells migrate and grow in other parts of the body, rather than with neoplastic changes *per se*. This process of migration, establishment and growth is an invasive process that involves the degradation of local extracellular matrix and angiogenesis [4, 5]. These observations suggest a mechanistic and may be even an evolutionary link between placentation type and malignancy rates and may explain in part why species develop malignancies in species-specific rates [6–8]. In this article, we review the mechanistic and evolutionary plausibility of such a link and conclude that a link may exist, but may be better explained by a positive pleiotropic effect between derived suppression of placental invasion in placental mammals and lower malignancy rates.

## HYPOTHESES

The cell biological similarity between placental invasion, in particular, the interstitial trophoblast invasion in humans and great apes, and metastasis of cancers and sarcomas, has motivated ideas about mechanistic and evolutionary links between these two biological phenomena (see Ref. [9] and references above). Here we use malignancy and metastasis interchangeably to refer to a situation where cancerous cells leave their location of primary growth and establish secondary tumors. The most widely held idea about the relationship between placentation and metastasis is that metastasis is a consequence of the evolution of invasive placentation. Here we review and evaluate this hypothesis and offer an alternative that relates placentation type to the likelihood of metastatic cancer.

### Antagonistic pleiotropy

**Figure 1.**
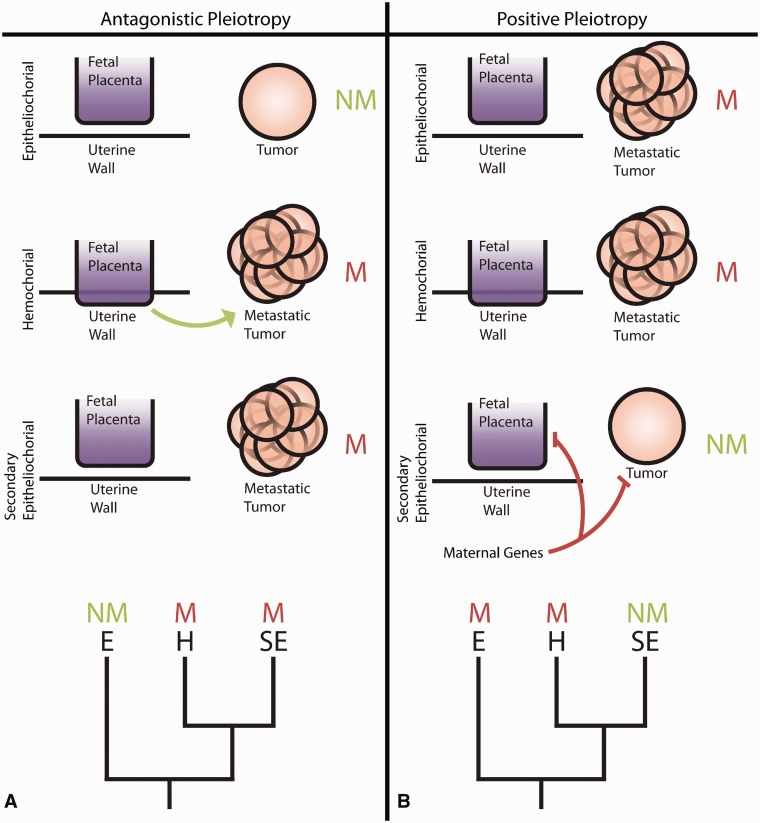
Two models for the relationship between placental invasiveness and malignancy. (**A**) The antagonistic pleiotropy model assumes that malignancy is a deleterious side effect of the evolution of invasive placentation. This model is supported by the similarity of the molecular mechanisms involved in trophoblast invasion and cancer invasion (metastasis). (**B**) The positive pleiotropy model assumes that the secondary loss of invasive placentation in some lineages of eutherian mammals is due to a derived maternal response to embryo attachment suppressing trophoblast invasion. These mechanisms are hypothesized to have a protective effect against invasiveness from cancers. *M* = metastatic cancer, *NM* = non-metastatic cancer, *E* = epitheliochorial placentation, *H* = hemochorial placentation, *SE* = secondarily epitheliochorial placentation

The similarity, at the molecular level, between placental invasiveness and metastatic invasiveness of tumors has inspired the idea that metastatic invasiveness is a consequence of the evolution of placental invasiveness [5, 9–12] (Fig. 1A). According to this hypothesis, metastatic cancer is a negative cost associated with the evolution of invasive placentation. Because placentation has a high early life fitness benefit and most cancers occur late in life, the consequence of metastatic cancer would be tolerable and invasive placentation would be selected [9, 13]. The selection against the negative pleiotropic effects due to metastatic cancer is expected to be weak since many cancers start late in life, often after the reproductive period [13]. In terms of phylogenetic patterns, this hypothesis predicts that metastatic cancers evolved after or coincidental with the evolution of invasive placentation (Fig. 1A).

The reasoning behind this argument is based on the shared molecular mechanisms of invasive placentation and metastasis. More specifically, special consideration is given to the processes that lead to angiogenesis, including the degradation of the extracellular matrix and expression of specialized adhesion molecules and more [5, 10, 11, 14].

If we follow the logic of the antagonistic pleiotropy model, then marsupials should not suffer metastatic cancers because they do not undergo invasive placentation at all and are derived from ancestors that also had non-invasive placentation. Thus the model predicts that masupials would not be subject to its antagonistic pleiotropic effects. In marsupials no truly invasive placentation has been described [15], even though the placentation in some marsupials has been described as ‘invasive’ [16], none of these cases show the hallmark of eutherian placentation, namely a direct interaction between invasive trophoblast cells and the endometrial stroma. In fact, after placentation in *Monodelphis* the density of endometrial stromal cells is very low, and the endometrium is almost completely occupied by glands and blood vessels [17]. Furthermore, invasive placentation evolved after the most recent common ancestor of marsupials and placental mammals, and thus, according to the antagonistic pleiotropy model, metastasis should also have evolved after the split between marsupials from the placental lineage. From these facts and from the logic of the antagonistic pleiotropy hypothesis, marsupials should have few or no metastatic tumors. However, several papers have shown that marsupials do get metastatic tumors, in particular for skin cancer [18–22]. Thus, metastasis originated prior to the origin of eutherian type invasive placentation.

The problem with the antagonistic pleiotropy hypothesis is that the molecular mechanisms shared between metastatic invasiveness and invasive placentation is not limited to these two processes. For instance, Murray [5] pointed out that these mechanisms are also shared with other biological processes, processes that are much older than invasive placentation, like wound healing. Furthermore, the shared mechanisms are also in common with a fundamental developmental process, the so-called epithelial to mesenchymal transition. This process is important for the development of at least all vertebrates, if not for the plurality of metazoans [23]. For these reasons we think that the antagonistic pleiotropy model is implausible, both on mechanistic and evolutionary grounds.

### Positive pleiotropy

Here we present an alternative hypothesis about the relationship between placental and metastatic invasiveness, based on the observation that the degree of invasiveness is, among eutherian mammals (=all species that derive from the most recent common ancestor of humans and armadillos and/or elephants), not predominantly determined by the physiology of the invasive trophoblast, i.e. the conceptus. Instead, invasiveness is determined by the nature of the maternal tissue, the endometrium. In short, the hypothesis is that, within placental mammals, the degree in placental invasiveness is positively correlated with the incidence of metastatic tumors, because the females of species with less invasive placentation have evolved mechanisms to suppress trophoblast invasion. We hypothesize that this derived capability of some mammalian lineages to suppress trophoblast invasion had a positive pleiotropic effect on the ability of the body to oppose metastatic invasion. In terms of the predicted phylogenetic pattern, the positive pleiotropy hypothesis predicts that metastatic cancer existed prior to the evolution of invasive placentation and persists in placental species with hemochorial placenta like humans. In contrast, in lineages with secondary epitheliochorial placentation (mostly hoofed animals and their relatives, like whales), malignancy was diminished (Fig. 1B).

The most broadly used classification of placental invasiveness is Grosser’s system [24] based on the tissue types at the fetal–maternal interface (see for instance [25]). The least invasive placentation is the so-called epitheliochorial placenta, where the placental chorion of the fetus is interfacing with the luminal epithelium of the uterine endometrium. Within placental mammals this situation is found in hoofed animals and their descendants (horses, cows and gazelles as well as whales and dolphins) and the lemurs [26]. The most invasive form of placentation is the hemochorial placentation, where the trophoblast erodes the luminal epithelium, part of the stroma and finally also the walls of the spiral arteries, so that the placental tissue is directly exposed to the maternal blood. An intermediate phenotype is the endotheliochorial placenta, where the fetus is intruding into the endometrium and establishing an intimate contact with the maternal blood vessels but does not breach their integrity. This type of placentation is for instance found in carnivores, e.g. cats and dogs.

While epitheliochorial placentation is typical for marsupials [16], the ancestral type for placental mammals is hemochorial, i.e. the most invasive type in Grosser’s classification [24, 27, 28]. This conclusion is based on molecular phylogenetic hypotheses about the interordinal relationships among eutherian mammals. A consequence of this model is that the less invasive placentation of equines, bovines and others is phylogenetically derived, contrary to a long held idea, which suggested that the situation in hoofed animals might be primitive. This means that the less invasive placentation in hoofed animals evolved from ancestors with more invasive placenta type, and this transition happened at least three times independently [28]. The question then is, what is the locus of evolutionary change responsible for the secondarily non-invasive placenta: changes that affect the trophoblast (a less aggressively invasive fetus) or changes that affect the endometrium (i.e. the mother has learned to keep the fetus out of her endometrium) or both? Some anecdotal evidence suggests that the derived condition is due to maternal adaptations rather than a less aggressive conceptus.

In humans and armadillos (i.e. species with a *uterus simplex*), implantation usually happens at the fundus of the uterus, where the endometrium is the thickest [29]. Nevertheless implantation can also happen at other locations, either in more distal regions of the uterus, where the endometrium is thinner, in the cervix or even in the body cavity [30, 31]. These ectopic pregnancies are very dangerous to the mother since unopposed trophoblast invasion can lead to unstoppable bleeding and eventual death. In the pig, a species with non-invasive placentation, ectopically implanted embryos are invasive [32], even though at their physiological implantation site they are unable to invade. This suggests that the evolution of secondarily non-invasive placentation is due to changes in the maternal endometrium that suppress the invasion of a conceptus that otherwise is still able to be invasive. This inference is further supported by the fact that the embryos of viviparous skinks, species that never evolved an invasive placenta, are also non-invasive when found ectopically [33].

From this data it seems that there are two phases of the evolution of invasiveness. One which occurred in the stem lineage of placental mammals, where the trophoblast evolved the ability to deeply invade the maternal tissue, and a second one where, in some eutherian lineages, the maternal endometrium evolved the capability to prevent the invasion by the trophoblast.

Given that the less invasive placenta evolved through maternal adaptations controlling the degree of invasiveness, it is possible that these mechanisms also have benefits with respect to the suppression of metastasis. The molecular mechanism of both, invasive placentation and metastasis, are very similar (see above), and thus derived mechanisms to suppress invasive placentation might also benefit the fight against invasive cancers. To test this idea we analyse some epidemiological data on animal malignancy in the next section as well as review data on gender differences in the survival rate after the diagnosis of metastatic cutaneous melanoma.

## CORRELATION BETWEEN PLACENTAL TYPE AND METASTATIC CANCER INCIDENCE

**Figure 2.**
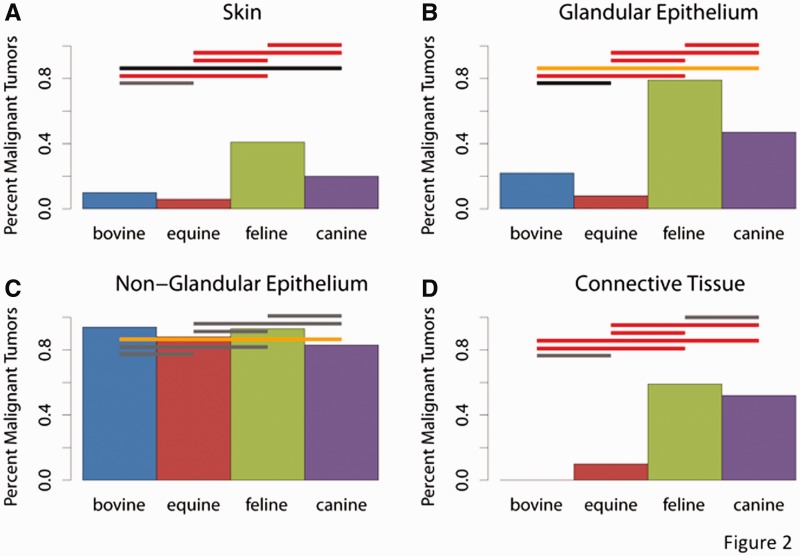
Percentage of tumors for each anatomic site and animal from Priester and Mantel [8]. The colored bars indicate significance values of pairwise Fisher’s exact tests. Gray is non-significant, black is *P* < 0.05, orange is *P* < 0.005 and red is *P* < 0.0001. Note that there are two patterns, one with uniformly high malignancy rates for non-glandular epithelial cancers, and one for the other categories of cancers. In the latter case the hoofed animals, which have non-invasive placentation, have lower malignancy rates than carnivores that have an invasive form of placentation

Here we test the prediction of the positive pleiotropy model that placental mammals with non-invasive placentation are less likely to develop metastatic cancers than species with more invasive placentation (Fig. 1B). We used the data from Priester and Mantel for analysis [8]. In their paper, the authors quantify tumor occurrence in four domestic species: bovines (cows), equines (horses), canines (dogs) and felines (cats) (Table 1; Fig. 2). In cooperation with 12 veterinary colleges, they collected data on over 200 000 animals and over 8500 tumors. They present the total number of malignant tumors in each domestic animal as a percentage of the total number of tumors collected. Of chief interest from their paper are their Figures 1 and 2 [8]. These figures illustrate rates of metastatic tumors in the four species-different anatomic sites.

**Table 1.** Frequency of metastatic cancer in four species at four anatomic sites

Anatomical location	Species	Malignant	Total	Malignancy rate
Skin	Cow	11	105	0.10
	Horse	26	437	0.06
	Cat	35	51	0.41
	Dog	338	1691	0.20
Glandular epithelium	Cow	10	45	0.22
	Horse	15	175	0.08
	Cat	45	57	0.79
	Dog	553	1184	0.47
Non-glandular epithelium	Cow	187	200	0.94
	Horse	119	136	0.88
	Cat	46	50	0.93
	Dog	263	316	0.83
Connective tissue	Cow	0	22	0.00
	Horse	13	127	0.10
	Cat	18	31	0.59
	Dog	323	626	0.52

Data from Priester and Mantel [8].

Bovines, equines, canines and felines are all eutherian mammals in the clade Laurasiatheria. Notably bovines and equines both have epitheliochorial placentation while canines and felines have endotheliochorial placentation. The prediction is that cows and horses should have lower metastatic cancer rates than cats and dogs in comparable forms of neoplastic tumors.

Since specific numbers of malignant tumors compared to the total were not given for all of the values that we analysed, we estimated the numbers by measuring the lengths of the malignancy bars in their figures and then rounded to the nearest whole number. To validate this method, we also estimated the numbers for known values (Table 1). The estimated numbers were always within two counts of values given in the article.

In the data from Priester and Mantel [8], there is a sufficient number of observations to analyse the malignancy rates of four categories of tumors: skin, glandular epithelial, non-glandular epithelial and connective tissue tumors. Estimated malignancy rates vary between zero percent in bovine connective tissue tumors (*N* = 22) and 94% in bovine non-glandular tumors (*N* = 200). We observe two types of patterns between species. One is found in non-glandular tumors where malignancy rates are uniformly high among all species (between 83 and 94%). In this category most pairwise comparisons are non-significant, except the comparison of bovine and canine malignancy rates (*P* = 6.0 × 10^−^^4^, Fisher’s exact test). Even though the latter comparison is statistically significant, it is doubtful that it reflects a biologically meaningful difference with both rates being quite high (83% in dog and 94% in cow).

The second pattern we found is one where malignancy rate are consistently higher in the two carnivore species than in cows and horses (Fig. 2). For instance in skin cancers, the average malignancy rate of hoofed animals is 6.8% while that for carnivores is 21%. For glandular epithelial cancers the rates are 11 and 48% and for connective tissue tumors it is 8.6 and 52%, respectively. This pattern is statistically highly significant and consistent with the predictions of the positive pleiotropy model, predicting that placental species with secondarily evolved non-invasive placenta gained a relative protection from invasive tumors.

## GENDER BIAS IN MELANOMA SURVIVAL RATE

If selection for maternal factors that limit the invasiveness of the trophoblast has a positive pleiotropic effect on the body’s resistance to invasive forms of cancer, one would expect that women are less vulnerable to invasive cancers than men. The idea is that the sex-specific selection during pregnancy can lead to the selection of genetic factors opposing invasiveness that are specifically expressed in females but not males. Testing this idea with epidemiological data is difficult, since the prediction is not about cancer incidence rate but about differences in disease progression. Remember that Lipizzaner horses have a very high incidence rate of melanoma and a very high survival rate. It is the latter that is relevant for our hypothesis.

Most epidemiological studies of cancer focus on incidence rate, since the aim is to understand the factors that cause cancer in the first place. Another frequently reported statistic is mortality rate which compounds incidence rate and disease progression. Mortality rate can only be used as indicating gender differences in disease progression if the incidence rate is in the opposite direction. For instance, Lens and Dawes [34] report data from UK, which shows a higher incidence rate of malignant melanoma in women during the years 1971-97 (their Figure 4; Ref. 34), but a lower mortality rate for women between 1991 and 2001 (their Figure 6). A similar situation has been reported by de Vries *et al.* [35] analysing data on malignant melanoma cases in Europe from 1953 to 1997. In their data men had generally lower incidence rate but higher mortality rates.

A better measure of gender differences in disease progression is the 5-year survival rate, since this measure is conditioned on the presence of a diagnosis of melanoma and is thus invariant with respect to incidence rate. Lens and Dawes [34] report on Scottish survival data that show a bias in favor of women. Overall the 5-year survival rate is 58% for males and 74% for females. The gender difference remains when the tumors are stratified by stage at the time of diagnosis.

A similar gender bias in survival rates in favor of females has been reported by Chang *et al.* [36] in 1998 in the National Cancer Data Base Report on cutaneous and non-cutaneous Melanoma. The analysis included US data from the years 1985 to -94. In their Table 5C the authors report a 5-year survival for Stage 0-I melanomas at diagnosis of 83.5% for males and 90.5% for females (*P* ≤ 10^−^^4^). For advanced-stage melanomas at diagnosis, the rates are 32.7% for males and 43.7% for females (*P* ≤ 10^−^^3^). Overall it seems that the female body is more capable to resist the spread and the effects of malignant cutaneous melanoma than the male body.

## DISCUSSION

Here we considered the popular model of antagonistic pleiotropy about the hypothesized relationship between invasive placentation and metastasic tumor incidence and found that it is not likely, given that the molecular similarities between them are shared with many much older biological processes like wound healing and epithelial–mesenchymal transitions during embryonic development. We offered an alternative model suggesting a positive pleiotropy between the maternal ability to suppress trophoblast invasion, as it occurs in some lineages of eutherian mammals like bovines and equines, and a lower incidence of metastatic tumors. A preliminary statistical analysis of cancer malignancy rates from cows, horses, cats and dogs is consistent with this hypothesis, at least for three of the four categories of tumors considered (skin cancer, glandular epithelial cancer and connective tissue sarcomas). The fourth category analysed (non-glandular epithelium) has very high malignancy rates among all four species.

Though these results are exciting, we are aware that our results are limited. Most importantly the data presented are not fully controlled for other potentially confounding variables. One is body size, horses and cows are large, cats and dogs are small and large animals have a higher risk of cancer due to the number of cells in their bodies than small animals. With higher risk of cancer the selective advantage of evolving antimetastatic mechanisms might be higher than in cats and dogs, irrespective of placentation type. Other factors are ecological and physiological. In the wild, hoofed animals spend more time exposed to direct sunlight than cats and dogs, although that is not the case for all members of the canine and feline clades (lions). Another physiological difference is diet, with hoofed animals being herbivorous and dogs and cats being carnivorous. In humans, meat consumption has been associated with higher cancer rates, and it might be that malignancy rate differences between herbivores and carnivores could be due to differences in diet. To be precise our analysis of the data assembled by Priester and Mantel [8] is a failed attempt at falsifying our hypothesis, rather than a conclusive proof, if such a thing does exists in the natural sciences.

At best our results could provide a jumping off point for future analysis. Further work is required to parse out the differences between metastatic rates across more species that vary among more dimensions than just placentation type. In order to do this, a cross-species database of metastatic rates is necessary. The study would ideally include several species from each placental category. To our knowledge such a database is either not publicly available or does not exist. But ultimately it will be necessary to experimentally test the positive pleiotropy hypothesis; for instance, with *in vitro* invasiveness assays that allows testing combinations of trophoblasts, cancer cells and endometrial cells of different species origins.

Our results suggest that the anatomic site of the neoplastic change heavily influences malignancy rates. There are high malignancy rates in all four species examined for non-glandular epithelial cancers in contrast to the pattern in other tumors. This suggests that mechanisms decreasing metastasis may be differentially expressed in different parts of the body.

The relationship between placentation and cancer metastasis could also plays a role in unravelling Peto’s paradox. Peto’s paradox highlights the puzzle that larger animals are able to survive despite their abundance of somatic cells and should be prone to tumor genesis and subsequent metastasis [37]. Large ferungulates like whales, horses and cows tend to have epitheliochorial placentation. Under the positive pleiotropic hypothesis, size increases would become more feasible because of the protective mechanisms associated with resisting placentation may have already existed prior to body size increase. This predicts that large body size should have evolved after the less invasive form of placentation, which is in broad agreement with the phylogenetic pattern of body sizes among ungulates.

Some large animals do not fall under this explanation. Elephants, for example, have vastly different mechanisms of placentation from ferungulates [26]. Consequently, they probably also have different mechanisms for metastatic cancer prevention. One possible explanation for this phenomenon is the large number of p53 family tumor suppressor gene copies present in elephants [37].

Aside from cross-species comparisons, human data can also be used to test the positive pleiotropy hypothesis. For instance, cases of preeclampsia (PE) could be an opportunity of study. PE is characterized by shallow implantation [38]. PE is a multifactorial disorder that can have a maternal contribution [39–43]. In cases where PE is caused by a genetic disposition in the mother, rather than in the conceptus, the positive pleiotropy model would predict that these patients also have a lower incidence of metastatic tumors. There is strong evidence that there is a negative relationship between PE and breast cancer risk [44, 45], although the relationship is not universal [46]. There are several candidate mechanisms to explain the protective (statistical) effect of PE, but it is not clear whether the factors causing shallow implantation are also the direct cause for the lower breast cancer risk [47, 48].

Gender differences may also play a role in protecting against metastasis, since females have to deal with uterine invasion while males do not. For that reason we reviewed some of the epidemiological literature on the survival rates with melanoma. There is good evidence that males die at higher rates due to melanoma even in populations where males have lower incidence rate. After a diagnosis of malignant melanoma, men survive at lower rates than women (see earlier for references). This pattern is consistent with the hypothesis that selection of factors that control invasive placentation can have a positive pleiotropic effect on cancer survival. In this context it is important to emphasize that the hypothesis does not make predictions about the incidence rate of cancers but about their progression to malignancy and death.

Gender differences in melanoma survival can have many causes. One factor that is discussed in the literature is the anatomical location of melanomas in men and women. Men have a higher frequency of melanomas on the trunk than women, and trunk melanomas have on average a worse outcome than melanomas on the limbs [49, 50]. Whether this factor alone is sufficient to explain the gender differences in survival rate is not clear.

Genetic mapping studies on cancer risk should also be analysed with possible pleiotropy to reproductive phenotypes in mind. Some published studies have found that differences in placental development and increased fecundity are both risk factors for metastatic cancer [51]. Determining whether these genes are directly connected to invasiveness of the placenta or differentially expressed across species with different placental types would be particularly interesting.

## CONCLUSION

The hypothesis for antagonistic pleiotropy is not supported due to the presence of metastatic cancer in marsupials and the use of shared mechanisms in other body functions such as wound healing. However, the link between invasive placentation and metastatic cancer within placental mammals seems plausible. Positive pleiotropy between degree of invasiveness, if controlled by the maternal tissues, and malignancy rate is a likely link. The results presented in this article give preliminary support to this hypothesis and suggest future studies of human genetic variation in this area.

## SUPPLEMENTARY DATA

Supplementary data is available at *EMPH* online.

Supplementary Data
