# Supplementary material for: Malignant cancer and invasive placentation: A case for positive pleiotropy between endometrial and malignancy phenotypes
Source: Evol Med Public Health. 2014 Oct 15;2014(1):136–45. doi: 10.1093/emph/eou022 (PMC4217742; doi:10.1093/emph/eou022)
Supplement: Supplementary Data [file supp_2014_1_136__index.html]

Malignant Cancer and Invasive Placentation: a case for positive pleiotropy between endometrial and malignancy phenotypes — Malignant cancer and invasive placentation — Supplementary Data 

# Malignant cancer and invasive placentation

## Supplementary Data

files

**Files in this Data Supplement:**

- Supplementary Data - pdf file
