# Supplementary material for: Malignant cancer and invasive placentation: A case for positive pleiotropy between endometrial and malignancy phenotypes
Source: Evol Med Public Health. 2014 Oct 15;2014(1):136–45. doi: 10.1093/emph/eou022 (PMC4217742; doi:10.1093/emph/eou022)
Supplement: Supplementary Data [file supp_eou022_supplemental.pdf]

1 Supplemental

Anatomic Site	Comparison	P-Value
Skin	bovine v equine	0.13
	bovine v feline	0.0001
	bovine v canine	0.0155
	equine v feline	0.0001
	equine v canine	0.0001
	feline v canine	0.0001
Glandular Epithelium	bovine v equine	0.0164
	bovine v feline	0.0001
	bovine v canine	0.0012
	equine v feline	0.0001
	equine v canine	0.0001
	feline v canine	0.0001
Non-Glandular Epithelium	bovine v equine	0.0783
	bovine v feline	0.7535
	bovine v canine	0.0006
	equine v feline	0.6013
	equine v canine	0.3209
	feline v canine	0.1418
Connective Tissue	bovine v equine	0.217
	bovine v feline	0.0001
	bovine v canine	0.0001
	equine v feline	0.0001
	equine v canine	0.0001
	feline v canine	0.5816

Table 1: **Significance for metastasis differences in four anatomic sites** This table gives the exact p-values for comparisons between species of malignant tumors at different anatomic sites. P-values lower than 0.0001 were rounded to 0.0001. Analysis was completed using the fisher.test function in the R Statistical Software. A two-tailed test was completed.
